# Click Synthesis of Triazole Polymers Based on Lignin-Derived Metabolic Intermediate and Their Strong Adhesive Properties to Cu Plate

**DOI:** 10.3390/polym15061349

**Published:** 2023-03-08

**Authors:** Yijie Jin, Manjusha Joshi, Takuma Araki, Naofumi Kamimura, Eiji Masai, Masaya Nakamura, Tsuyoshi Michinobu

**Affiliations:** 1 Department of Materials Science and Technology, Tokyo Institute of Technology, 2-12-1 Ookayama, Meguro-ku, Tokyo 152-8552, Japan; 2 Department of Forest Resource Chemistry, Forestry and Forest Products Research Institute, Tsukuba 305-8687, Japan; 3 Department of Materials Science and Bioengineering, Nagaoka University of Technology, Nagaoka 940-2188, Japan

**Keywords:** adhesive, biomass, click chemistry, lignin, triazole polymer

## Abstract

2-Pyrone-4,6-dicarboxylic acid (PDC) is a chemically stable metabolic intermediate of lignin that can be produced on a large scale by transforming bacteria. Novel biomass-based polymers based on PDC were synthesized by Cu(I)-catalyzed azide-alkyne cycloaddition (CuAAC) and fully characterized by nuclear magnetic resonance, infrared spectroscopies, thermal analysis, and tensile lap shear strength measurements. The onset decomposition temperatures of these PDC-based polymers were all above 200 °C. In addition, the PDC-based polymers exhibited strong adhesive properties to various metal plates, with the highest adhesion to a copper plate of 5.73 MPa. Interestingly, this result was in contrast to our previous findings that PDC-based polymers weakly adhere to copper. Furthermore, when bifunctional alkyne and azide monomers were polymerized in situ under hot-press conditions for 1 h, the resulting PDC-based polymer displayed a similar adhesion to a copper plate of 4.18 MPa. The high affinity of the triazole ring to copper ions improved the adhesive ability and selectivity of the PDC-based polymers to copper while still maintaining the strong adhesive ability to other metals, which is conducive to enhancing the versatility of PDC-based polymers as adhesives.

## 1. Introduction

To realize a carbon neutral society, renewable materials, such as biomass-derived polymers, have attracted significant attention as alternatives to petrochemical products [1,2,3,4,5,6,7,8]. The production of (pseudo-)aromatic functional small molecules from biomass is, thus, very important. Lignin is one of the most promising carbon resources, as it is an aromatic biomass with a three-dimensional reticular structure that is abundant in nature. Although lignin can be broken down into lower-molecular-weight compounds, only a small fraction of the formed products is utilized for the synthesis of aromatic chemicals [9,10,11,12]. In nature, however, there are a variety of microorganisms that can degrade lignin and convert it into its energy source through complex metabolic pathways [13,14,15,16,17,18]. In a previous study, lignin-derived aromatic compounds were successfully converted into 2-pyrone-4,6-dicarboxylic acid (PDC) on a large-scale using engineered *Pseudomonas putida* strains [19]. PDC consists of a polar pseudo-aromatic pyrone ring with two carboxyl groups, which are unstable under alkaline conditions [20]. Because biomass-based polymers have great potential to replace petrochemicals, it is important to develop PDC-based polymers with unique functions and superior properties. We have previously demonstrated that PDC undergoes condensation with diols to synthesize a variety of PDC polyesters, which exhibit good biodegradability and mechanical properties [21,22,23,24,25,26,27]. In addition, PDC-based polyesters are excellent adhesives with strong adhesive properties for a wide range of metals or non-metallic materials, such as glass [22,28,29]. However, the high adhesive properties of PDC-based polyesters were mainly observed for iron, aluminum, and stainless steel, while copper was not a suitable metal in the previous adhesive experiments. In order to reveal the structure–property relationship and develop versatile PDC-based polymeric adhesives, it is necessary to improve the adhesive properties of PDC-based polymers to copper.

Esterification is one of the most convenient and useful polymerization methods in industry. However, other efficient polymerization methods have also been desired for the synthesis of PDC-based polymers to scrutinize the chemical structure–polymer property relationship. Click chemistry is a concept in organic synthesis that is used to synthesize functional polymers [30]. Among the many click chemistry reactions (or click reactions), Cu(I)-catalyzed azide-alkyne cycloaddition (CuAAC) has high efficiency and applicability to various classes of compounds, producing 1,4-disubstituted triazole derivatives [31,32,33,34,35]. We preliminarily reported that PDC-based polymers can be synthesized by CuAAC without using basic amines as a ligand for the Cu(I) catalyst [28]. However, the diazide monomer used was found to be explosive due to the nitrogen-containing azide group and cannot be mass produced on an industrial scale. Although PDC-based polymers generally exhibit strong adhesion to a wide range of metals and glasses, not many comprehensive studies about the adhesive properties of PDC-based polymers synthesized by CuAAC have been reported. As it is well-known that copper ions strongly interact with triazole rings [36], efforts to synthesize PDC-based polytriazoles and investigate their adhesive properties to copper plates have continued. In this study, PDC-based polymers were synthesized by the ligand-free CuAAC polymerization of PDC-dialkyne and oligo(ethylene glycol) diazides, and their adhesive properties to metal plates were evaluated. Moreover, PDC-dialkyne and oligo(ethylene glycol) diazides were polymerized in situ between metal plates under hot-press conditions, which demonstrated sufficiently high adhesive properties to the copper plates. Specifically, our main contributions are summarized as follows:

(1) We synthesized PDC polymers by ligand-free CuAAC polymerization using PDC-dialkyne and oligo(ethylene glycol) diazides and fully characterized them by NMR, IR spectroscopy, gel permeation chromatography, and thermal analysis. We discussed the effect of different polymerization conditions on the molecular weight and yield of the polymers.

(2) In addition, we evaluated the adhesive properties of PDC-based triazole polymers to metal plates by tensile lap shear strength measurements. We discussed the relationship between adhesive properties and metal type in terms of the chemical structure of the polymer main chains.

(3) Lastly, we, for the first time, studied the in situ polymerization of PDC-dialkyne and oligo(ethylene glycol) diazides between metal plates under hot-press conditions at different temperatures and evaluated their adhesive properties to different metal plates. We also discussed the reason why the developed polymers exhibit different adhesive properties at different hot-press temperatures.

## 2. Materials and Methods

### 2.1. Materials

2-Pyrone-4,6-dicarboxylic acid (PDC) was prepared from vanillic acid using an engineered *P. putida* strain, as reported previously [19]. Propargyl alcohol was purchased from Kanto Chemical Co., Inc. (Tokyo, Japan). 1,11-Diazido-3,6,9-trioxaundecane (**2**), 1,17-diazido-3,6,9,12,15-pentaoxaheptadecane (**3**), and copper(I) iodide were purchased from Sigma-Aldrich, Co. (St. Louis, MI, USA). *N*,*N*′-Dicyclohexylcarbodiimide (DCC) and 4-dimethylaminopyridine (DMAP) were purchased from Tokyo Kasei Kogyo Co., Ltd. (Tokyo, Japan). All reagents were used without further purification.

### 2.2. Measurements

Nuclear magnetic resonance (^1^H-NMR and ^13^C-NMR) spectra were recorded on a JEOL (Tokyo, Japan) model 400YH spectrometer at 20 °C. All chemical shifts are reported in parts per million downfield from SiMe_4_, using the solvent’s residual signal as an internal reference. The resonance multiplicity was described as s (singlet), d (doublet), t (triplet), and m (multiplet). Fourier transform infrared spectra (FT-IR) were recorded on a JASCO (Tokyo, Japan) FT/IR-4200 spectrometer. Gel permeation chromatography (GPC) was measured at 40 °C using a JASCO HSS-1500 system with a refractive index (RI) detector. DMF with lithium bromide (5 mM) was used as the eluent at the flow rate of 0.6 mL min^−1^. Thermogravimetric analysis (TGA) and differential scanning calorimetry (DSC) measurements were recorded under a nitrogen flow on a Rigaku (Tokyo, Japan) Thermo plus TG8120 and DSC8230, respectively, at a heating rate of 10 °C min^−1^, from 20 °C to 500 °C for TGA and from −50 °C to 200 °C for DSC.

Tensile lap shear strength measurements were taken according to JIS K 6850-1994 with a Shimadzu Co. (Tokyo, Japan) autograph AGS-10kNX STD. The size of the metal plate pieces (copper or iron) used for the adhesion measurements was 25.0 mm × 100.0 mm × 1.6 mm. Before their use, the metal plates were washed under sonication with a series of solvents, such as dichloroethane, acetone, methanol, and DI water, and were then surface treated with a Technovision Inc. (Saitama, Japan) UV-O_3_ Cleaner UV-208 for 300 s. Approximately 30 mg of the PDC polymer (**P1** or **P2**) solid or a solution of PDC-dialkyne (**1**) and 1,11-diazido-3,6,9-trioxaundecane (**2**)/1,17-diazido-3,6,9,12,15-pentaoxaheptadecane (**3**) (1:1 mol/mol, 15.5 mg (**1**):13 μL (**2**)/13.2 mg (**1**):15 μL (**3**)) was cast on the metal plate surface. A set of two plates was placed in contact with each other by hot pressing at 10 MPa and cured for one hour under vacuum. In order to control the reliability of the adhesive conditions in each test environment, a 0.20 mm thick Teflon spacer was used between the metal plates. This was used to keep the thickness of the adhesive layer at 0.20 ± 0.02 mm. In order to eliminate residual stresses within the adhesive itself during each measurement, the metal plates and the adhesive were slowly cooled to room temperature after hot pressing. The failure force was measured at room temperature at a rate of 1 mm min^−1^. In order to balance the forces, an additional set of metal plates was used when measuring the tensile lap shear strengths (Figure S1). In addition to the two original metal plates adhered by the adhesive, a pair of other metal plates with the same size and thickness was placed on each side to fill the shortfall and balance the force on the measured metal plates. The adhesive strengths were calculated as the failure force divided by adhesive area (25.00 ± 1.00 mm × 10.00 ± 1.00 mm).

### 2.3. Synthesis

Diprop-2-ynyl 2-oxo-2H-pyran-4,6-dicarboxylate (**1**): PDC (1.84 g, 10.0 mmol), propargyl alcohol (1.47 mL, 25.0 mmol), and *N*,*N*-dimethylaminopyridine (244 mg, 2.00 mmol) were taken in a two-necked round bottom flask and 1,2-dimethoxyethane (50 mL) was added under argon. After cooling to 0 °C, dicyclohexylcarbodiimide (4.54 g, 22.0 mmol) was added, and the reaction mixture was warmed to 20 °C and stirred for 16 h. The precipitates were filtered off, and the filtrate was washed with saturated aqueous NaCl solution and DI water and dried over Na_2_SO_4_. Evaporation of the solvents furnished a crude product, which was purified by column chromatography (SiO_2_, chloroform/ethyl acetate 20:1) and dried in vacuo, which afforded **1** (1.31 g, 50.5%) as a white solid.

^1^H NMR (400 MHz, DMSO-d_6_, 293 K): δ = 7.24 (d, J = 1.11 Hz, 1 H), 7.11 (d, J = 1.05 Hz, 1 H), 4.96–4.95 (m, 4 H), 3.70–3.68 (m, 2H); ^13^C NMR (100 MHz, DMSO-d_6_, 293 K) δ = 162.16, 159.82, 158.47, 148.65, 142.48, 123.40, 108.80, 79.52, 77.96, 54.64, 54.45; IR (neat): ν = 3283, 3100, 2130, 1725, 1641, 1562, 1436, 1416, 1400, 1375, 1327, 1228, 1167, 1110, 1087, 987, 950, 936, 889, 758, 685, 647 cm^−1^.

**P1**: 1,11-Diazido-3,6,9-trioxaundecane (**2**) (0.416 mL, 2.00 mmol), copper(I) iodide (5 mol%, 38 mg, 0.20 mmol), and **1** (520 mg, 2.00 mmol) were taken in a round-bottom flask, and dehydrated DMF (3 mL) was added. After stirring at 20 °C for 70 h, the reaction mixture was poured into CH_3_OH (300 mL); the precipitate formed was filtered, thoroughly washed with CH_3_OH, and then dried in vacuo to afford **P1** (424.6 mg, 42.1%) as a dark orange solid.

^1^H NMR (400 MHz, DMSO-d_6_, 293 K): δ = 8.17 (d, J = 3.42 Hz, 2n H), 7.24 (d, J = 1.08 Hz, n H), 7.02 (d, J = 1.08 Hz, n H), 5.35 (s, 4n H), 4.49–4.46 (m, 4n H), 3.76–3.73 (m, 4n H), 3.46–3.42 (m, 4n H), 3.39–3.36 (m, 4n H); IR (neat): ν = 3142, 3095, 3007, 2871, 1732, 1644, 1559, 1455, 1446, 1418, 1396, 1348, 1325, 1241, 1168, 1112, 1051, 998, 953, 833, 755, 702, 664 cm^−1^.

**P2**: 1,17-Diazido-3,6,9,12,15-pentaoxaheptadecane (**3**) (0.294 mL, 1.00 mmol), copper(I) iodide (5 mol%, 19 mg, 0.10 mmol), and **1** (260 mg, 1.00 mmol) were taken in a round-bottom flask, and dehydrated DMF (2 mL) was added. After stirring at 20 °C for 70 h, the reaction mixture was filtered off, and the filtrate was poured into CH_3_OH (300 mL). The precipitate was filtered, thoroughly washed with CH_3_OH, and then dried in vacuo to afford **P2** (220.8 mg, 37.3%) as a dark brown solid.

^1^H NMR (400 MHz, DMSO-d_6_, 293 K): δ = 8.20 (d, J = 3.63 Hz, 2n H), 7.19 (s, n H), 7.03 (s, n H), 5.37 (s, 4n H), 3.40 (s, 8n H), 4.50–4.44 (m, 4n H), 3.77–3.74 (m, 4n H), 3.47–3.44 (m, 8n H), 3.41–3.38 (m, 8n H); IR (neat): ν = 3096, 2915, 2870, 1734, 1683, 1670, 1652, 1636, 1558, 1540, 1520, 1507, 1488, 1472, 1456, 1418, 1396, 1348, 1325, 1245, 1113, 1051, 1033, 951, 835, 759, 719, 703, 670, 658, 649 cm^−1^.

## 3. Results

### 3.1. Polymer Synthesis and Characterization

**Scheme 1:**
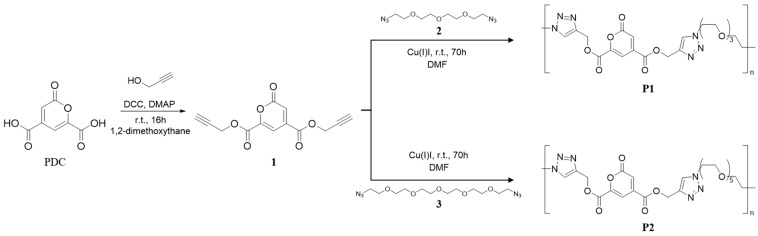
Synthetic routes of **P1** and **P2**.

In order to apply the CuAAC, it is necessary to convert the carboxylic acid functionality of PDC into either an alkyne or azide. Because a multi-step synthesis is required and the chemical stability of the azide-bearing PDC is unknown, the construction of azide groups is challenging. We thus selected PDC-alkyne **1** and synthesized it by the esterification reaction between PDC and propargyl alcohol (Scheme 1). By using coupling reagents, dicyclohexylcarbodiimide (DCC) and *N*,*N*-dimethylaminopyridine (DMAP), the carboxylic acid groups of PDC were successfully converted into the terminal alkynes in a 50.5% yield. The impurities of this reaction were soluble in hexane, while PDC-alkyne **1** was insoluble. Therefore, the purity of **1** was improved by washing with hexane after column chromatography. The high purity of monomers usually increases the polymerization yield and the molecular weights of the target polymers. The click polymerization of **1** with equimolar amounts of 1,11-diazido-3,6,9-trioxaundecane (**2**) or 1,17-diazido-3,6,9,12,15-pentaoxaheptadecane (**3**) in DMF, at room temperature, was Cu(I)-catalyzed to give the corresponding PDC-based polytriazoles, **P1** and **P2**, in ~40% yields. Both polymers were soluble in polar nonprotic solvents, such as DMF and DMSO, but were only slightly soluble in chloroform and insoluble in acetone and methanol.

**Figure 1:**
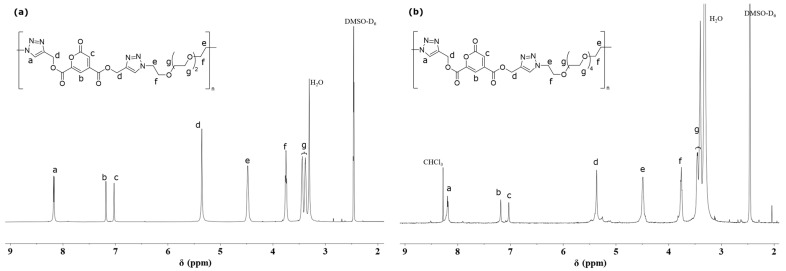
^1^H NMR spectra of (**a**) **P1** and (**b**) **P2** in DMSO-d_6_ at 20 °C.

The chemical structures of the resulting triazole polymers were characterized by spectroscopic measurements. The IR spectra of both polymers showed the absence of the azide peak and C≡C peak at around 2110 cm^−1^ and 2130 cm^−1^, respectively, indicating that both the alkyne monomer **1** and azide comonomer were consumed (Figure S4). The ^1^H-NMR spectra of **P1** and **P2** were measured in DMSO-d_6_ at 20 °C (Figure 1). The terminal alkyne peak of **1** at 3.69 ppm disappeared, and a new peak ascribed to the triazole ring proton appeared at ~8.2 ppm. In addition, the aromatic protons ascribed to the PDC pyrone ring were detected at almost the same position as those of the monomer. These results indicated that the **P1** and **P2** polymers were successfully formed. The integration ratios of the pyrone ring protons and oligo(ethylene glycol) chain protons supported the successful alternating copolymerization. Some protons were observed to have multiple peaks due to the asymmetry of the PDC structure. The number-average molecular weight (M_n_) and polydispersity index (M_w_/M_n_), determined by GPC with the eluent of DMF containing 5 mM LiBr at 40 °C, were 2500 and 1.12 for **P1** and 2300 and 1.18 for **P2**, respectively.

Polymerization conditions were comprehensively investigated by using **P2**. In the presence of basic amines as a ligand for the Cu(I) catalyst, different results were obtained with different catalysts, but the yields were always low. For example, when Cu(I)Br was used as the catalyst and *N*,*N*,*N*′,*N*″,*N*″-pentamethyldiethylenetriamine (PMDETA) as the ligand, the yield after 48 h reaction at 60 °C in DMF was about 16%, and the M_n_ and M_w_/M_n_ were 1800 and 1.12, respectively. When the polymerization was performed in the presence of Cu(I)Br but without any basic amine ligands, the yield improved to 40%. This result suggested that the use of different Cu(I) catalysts did not have a noticeable impact on the polymerization yield. Polymerization at different temperatures produced similar yields. Finally, polymerization time was investigated. When polymerization was performed at room temperature, the yield of **P2** gradually increased with the polymerization time and leveled off after 48 h. Overall, the polymerization conditions shown in Scheme 1 were optimized.

### 3.2. Thermal Properties

**Figure 2:**
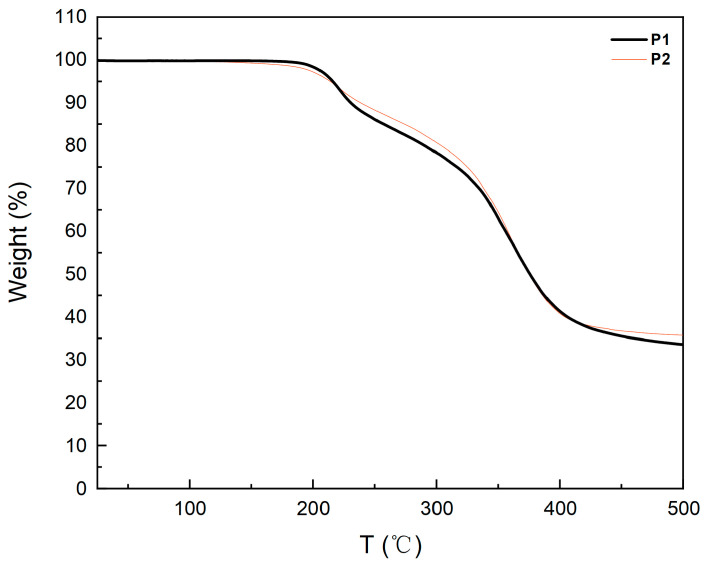
TGA curves of **P1** and **P2** at the heating rate of 10 °C min^−1^ under flowing nitrogen.

The thermal stability of the triazole polymers was investigated by thermogravimetric analysis (TGA). Decomposition of both **P1** and **P2** proceeded in two steps with the onset decomposition temperatures of 216 and 213 °C, respectively (Figure 2). The weight loss of **P1** and **P2** at about 210–220 °C may be caused by the decomposition of the pyrone ring of the PDC unit [22]. It has been reported that PDC has adhesive properties to various metals based on the ring opening of the pyrone ring, followed by the covalent bond formation with the metal surface at around 180–190 °C [22,37]. Based on this fact, both **P1** and **P2** have the potential for use as adhesives. Differential scanning calorimetry (DSC) measurements of **P1** and **P2** showed that there were no crystallization and melting peaks in the temperature range of −50 to 200 °C.

### 3.3. Adhesive Properties

It has previously been demonstrated that PDC-containing polyesters and epoxy resins have strong adhesion to metals and glass surfaces [22,37]. For example, PDC polyesters, prepared by the polycondensation between PDC, bis(2-hydroxyethyl)terephthalate, and bis(2-hydroxyethyl) PDC, generally displayed higher adhesions to Al, Fe, and SUS plates than brass and Cu plates. However, it is well known that the triazole ring has specific adhesive properties for copper ions [38]. It is thus interesting to study the adhesive properties of the PDC-based triazole polymers. In this context, the tensile strain of **P1** and **P2** was tested for iron and copper plates as representatives of high adhesion and low adhesion metals of PDC polyesters, respectively. In this study, we also tested the in situ polymerization under hot-press conditions to provide adhesion, and the results were compared with those of **P1** and **P2**.

**Figure 3:**
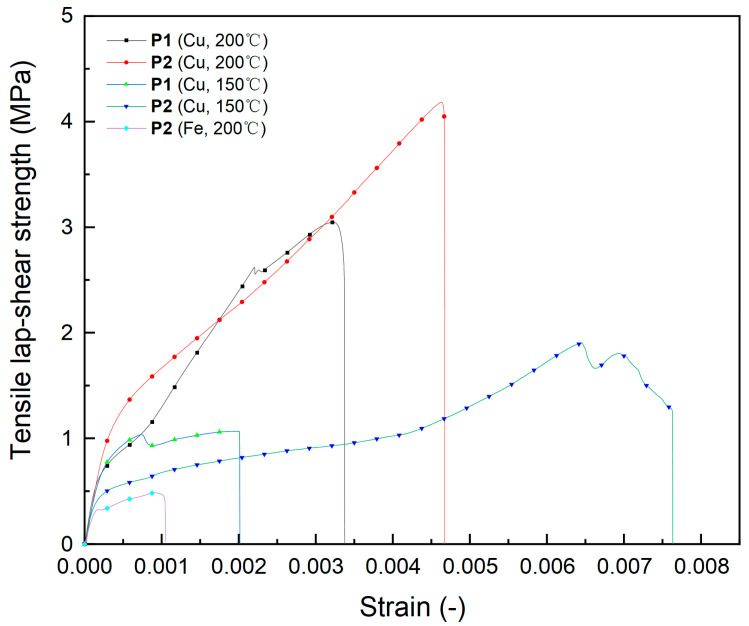
Adhesive properties of the in situ polymerized **P1 (1 + 2)** and **P2 (1 + 3)** on copper (Cu) and iron (Fe) plates under different conditions (at 150 °C or 200 °C for one hour).

First, the in situ polymerization was tested (Figure S5). It is known that thermal cycloaddition between the terminal alkyne and azide groups can occur, but the products are regio-isomeric mixtures of the 1,4-triazole and 1,5-triazole derivatives, and the yields are usually low in the absence of the appropriate catalysts [39]. Equimolar amounts of PDC dialkyne **1** and oligo(ethylene oxide) diazide **2** or **3** (a total amount of 30 mg) were mixed and the solution was cast on the surface of the metal plates. The paired plates were then hot pressed under vacuum at the pressure of 10 MPa and different temperatures for one hour to allow for the in situ polymerization, which directly exhibited adhesion to the metal surfaces. Tensile lap shear strength measurements were performed on the iron and copper plates according to the JIS K 6850-1994 testing method. It was found that no adhesion occurred when the hot-press temperature was lower than 150 °C or higher than 250 °C. Previous surface observations from ESCA measurements revealed that the attack of the metal surface hydroxyl or oxide groups on the PDC rings leads to ring-opening reactions and covalent bond formation between the metal surface and the PDC-based polymers [40]. Therefore, the PDC ring unit was thermally stable at temperatures lower than 150 °C, and no adhesion occurred. On the other hand, heating to 250 °C resulted in a partial carbonization unsuitable for producing adhesive properties. For the copper plates, the tensile lap shear strengths of **P1 (1 + 2)** and **P2 (1 + 3)** prepared at 200 °C were higher than those of prepared at 150 °C (Figure 3 and Table 1). This indicated that the ring-opening reaction of the PDC pyrone ring proceeded at nearly 200 °C. The maximum tensile lap shear strengths of **P1 (1 + 2)** and **P2 (1 + 3)** prepared at 200 °C were 3.05 MPa and 4.18 MPa, respectively. The longer oligo(ethylene glycol) chain as a monomer led to the higher strength. This finding indicated that the adhesive property originates not only from the PDC density but also from the other components of the entire polymer. When the different metal plates were compared, the adhesion ability of **P2 (1 + 3)** to the copper plate was much greater than that to the iron plate under the same conditions. For example, **P1 (1 + 2)** displayed no adhesion to iron plates under any condition. As previously suggested by Finn et al., this may be attributed to both the catalytic effect of a small amount of leached Cu(I) on the surface of the copper plate and the interaction between copper and the triazole ring [41].

**Figure 4:**
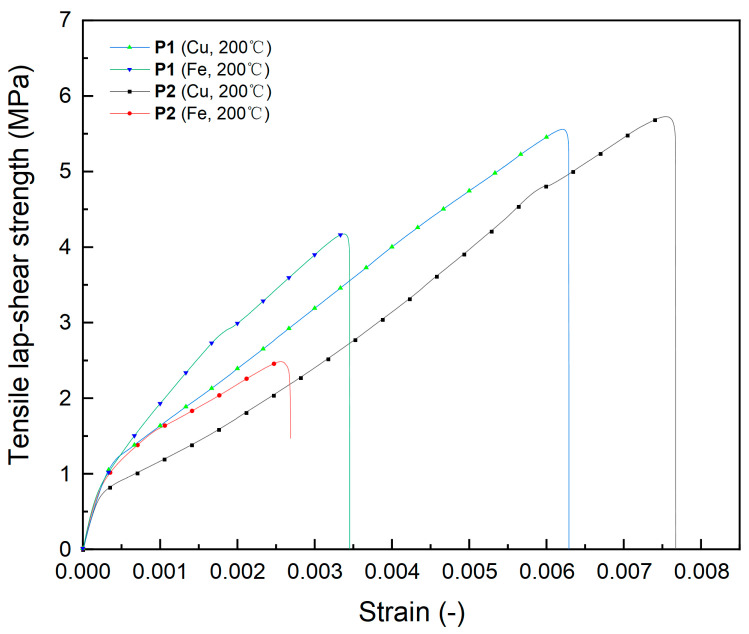
Adhesive properties of **P1** and **P2** hot pressed at 200 °C for one hour between copper (Cu) and iron (Fe) plates.

**P1** and **P2** were prepared by CuAAC polymerization at room temperature for 70 h (Scheme 1). These polymers were also employed for the adhesion tests to both copper and iron plates (Figure S6). After 30 mg of the **P1** or **P2** solid powder was uniformly cast on the surface of the metal plates, the paired plates were hot pressed under vacuum at 10 MPa and 200 °C for one hour. **P1** and **P2** exhibited higher adhesion strengths than the in situ polymerized samples on the metal plates (Figure 4 and Table 2). The maximum tensile lap shear strengths of **P1** for the copper and iron plates were 5.56 MPa and 4.17 MPa, respectively; those of **P2** were 5.73 MPa and 2.48 MPa, respectively. These results support that the thermal in situ polymerization of **1** and **2**/**3** was catalyzed by the Cu(I) ions leaked from the metal plate. These results also imply that the thermal in situ polymerization of **1** and **2**/**3** may not have been completed and that extending the hot-pressing time would have further improved the adhesive properties. The adhesion ability of **P1** was stronger than that of **P2** for the iron plates, while both polymers possessed almost the same adhesion ability to the copper plates. The adhesion of **P1** and **P2** to the copper plates, whether they are in situ polymerized, or not, was stronger than that to the iron plates. The previously reported adhesive properties of PDC-based polyesters without triazole rings to copper were weaker than to other metals [22], while the result in this study was found to be contrary to the previously reported adhesive properties of PDC-based polyesters. Thus, it was concluded that the triazole ring introduced by the CuAAC click reaction improved the interaction between the PDC-based polymers and the copper plate surface and substantially enhanced the adhesive properties of the PDC-based polymer to copper.

## 4. Conclusions

PDC polyesters containing the triazole rings in their main chain backbone were successfully synthesized by CuAAC polymerization without the use of basic amines as ligands. The tensile lap shear strength measurements indicated that the PDC-based triazole polymers exhibited strong adhesion to the metal plates. Attributed to the strong specific interaction of the triazole ring with the copper ions, the polymers showed a significantly higher adhesion to the copper plates than to the iron plates, with a maximum tensile lap shear strength of 5.73 MPa under the measurement conditions. It was also demonstrated that the alkyne- and azide-functionalized bifunctional monomers could be polymerized in situ on the copper plates by hot pressing, thus producing a sufficient adhesion. This process is more straightforward than the process of polymerization followed by adhesion. In today’s manufacturing industry, represented by the automobile industry, adhesives that can strongly adhere to different materials are often required. In our previous study, most of the PDC-based polymers had slightly weaker adhesion to copper than to other metals, such as iron, aluminum, and stainless steel. Therefore, the improved adhesion of PDC-based polymers to copper will enhance the versatility of PDC-based polymers as practical adhesives.

Further optimization of the chemical structure is expected to improve the adhesive properties and expand the scope of the materials. In this study, we demonstrated that the adhesive property of PDC-based polymers to copper can be improved by using the triazole structure. However, the overall adhesive performance is yet to be perfectly optimized. As already described, revealing the chemical structure–property relationship is the key to achieving high-performance adhesives based on PDC-based polymers. In this context, the comonomer structure, i.e., the type of diazide, needs to be further optimized. In other words, we think that the overall adhesive properties of PDC-based polymers to various materials can be further improved, making them more versatile materials. This will also contribute to the industrial utilization of lignin as a fine chemical.

## Figures and Tables

**Table 1:** Adhesion conditions and tensile lap- hear strengths (MPa) of the in situ polymerized **P1** and **P2** according to JIS K 6850-1994.

	Tensile Lap Shear Strength (MPa)			
	100 °C	150 °C	200 °C	250 °C
**P1 (1 + 2)**-Cu	-	1.06	3.05	-
**P1 (1 + 2)**-Fe	-	-	-	-
**P2 (1 + 3)**-Cu	-	1.90	4.18	-
**P2 (1 + 3)**-Fe	-	-	0.48	-

**Table 2:** Tensile lap shear strengths (MPa) of **P1** and **P2** hot pressed at 200 °C for one hour according to JIS K 6850-1994.

	Tensile Lap Shear Strength (MPa)
**P1**-Cu	5.56
**P2**-Cu	5.73
**P1**-Fe	4.17
**P2**-Fe	2.48

## Data Availability

The data presented in this study are available on request from the corresponding author.

## Supplementary Materials

The following supporting information can be downloaded at https://www.mdpi.com/article/10.3390/polym15061349/s1: Figure S1: Tensile lap shear strength measurements according to JIS K 6850-1994; Figure S2: ^1^H NMR spectrum of **1** in DMSO-d_6_ at 20 °C; Figure S3: ^13^C NMR spectrum of **1** in DMSO-d_6_ at 20 °C; Figure S4: Infrared spectra of (a) 1, (b) P1, and (c) P2; Figure S5: The tensile lap shear strength measurements of the in situ polymerized P1 (1 + 2) and P2 (1 + 3); Figure S6: The tensile lap shear strength measurements of P1 and P2 prepared by CuAAC polymerization.
