# Supplementary material for: Click Synthesis of Triazole Polymers Based on Lignin-Derived Metabolic Intermediate and Their Strong Adhesive Properties to Cu Plate
Source: Polymers (Basel). 2023 Mar 8;15(6):1349. doi: 10.3390/polym15061349 (PMC10051500; doi:10.3390/polym15061349)
Supplement: Supplementary file 1 [file polymers-15-01349-s001.zip › polymers-2255199-supplementary.pdf]

Supporting Information

Click Synthesis of Triazole Polymers Based on Lignin-Derived Metabolic Intermediate and Their Strong Adhesive Properties to Cu Plate

Yijie Jin [1], Manjusha Joshi [1], Takuma Araki [2], Naofumi Kamimura [3], Eiji Masai [3], Masaya Nakamura [2] and Tsuyoshi Michinobu [1,*]

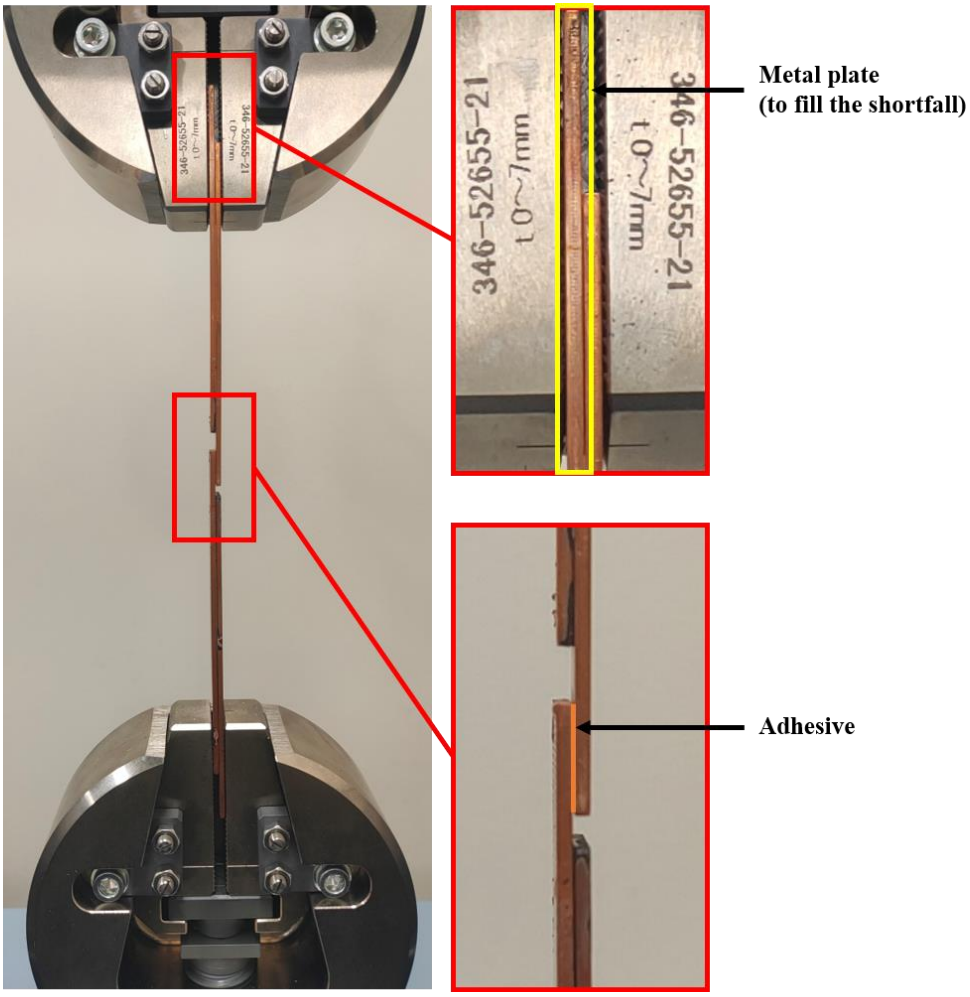

Figure S1. Tensile lap-shear strength measurements according to JIS K 6850-1994.

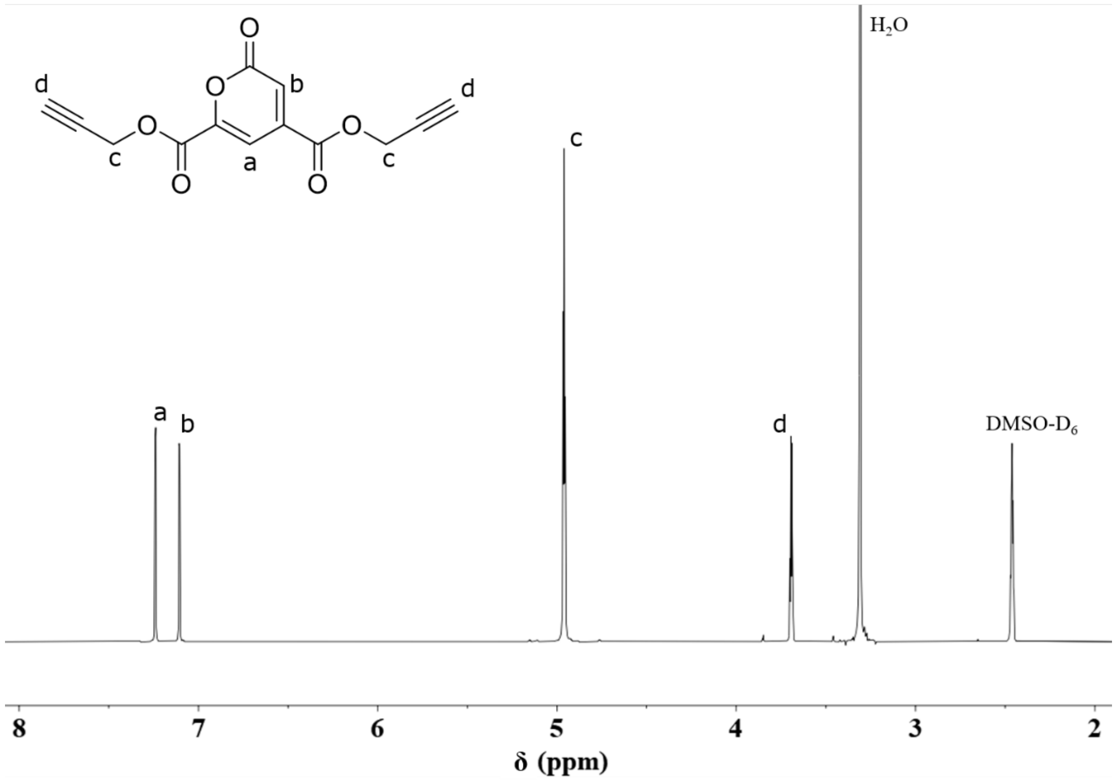

Figure S2. ^{1}H NMR spectra of **1** in DMSO-d$_6$ at 20 °C.

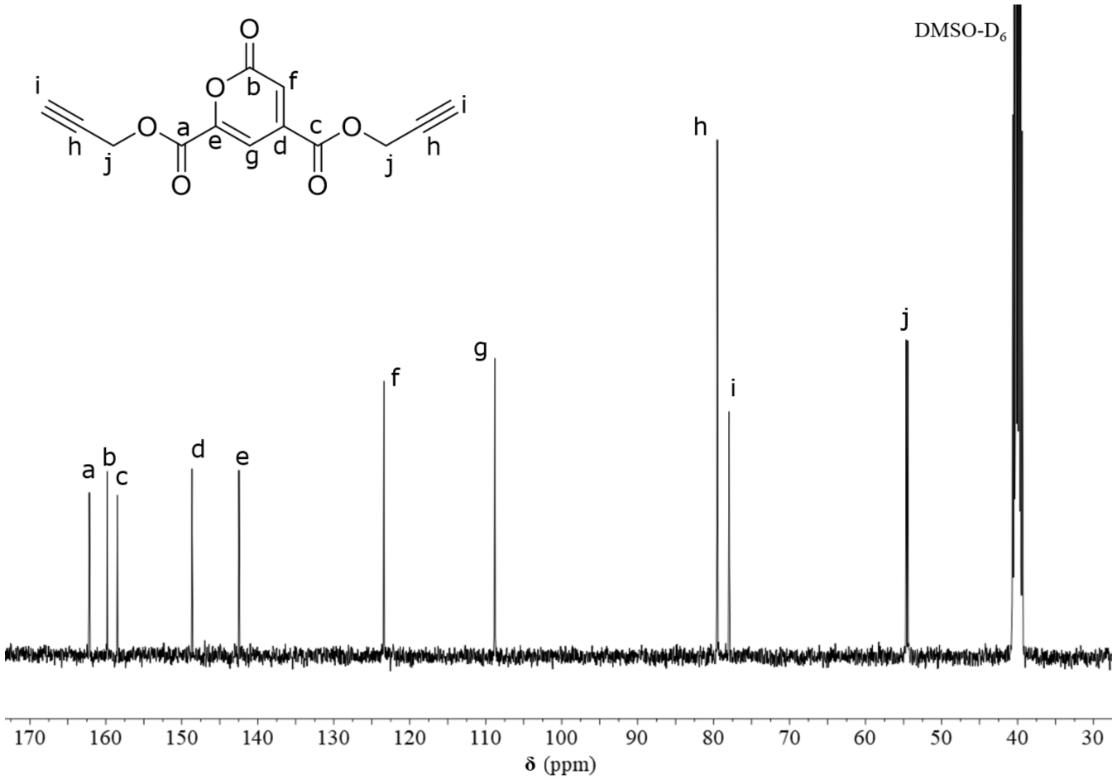

Figure S3. ^{13}C NMR spectra of **1** in DMSO-d$_6$ at 20 °C.

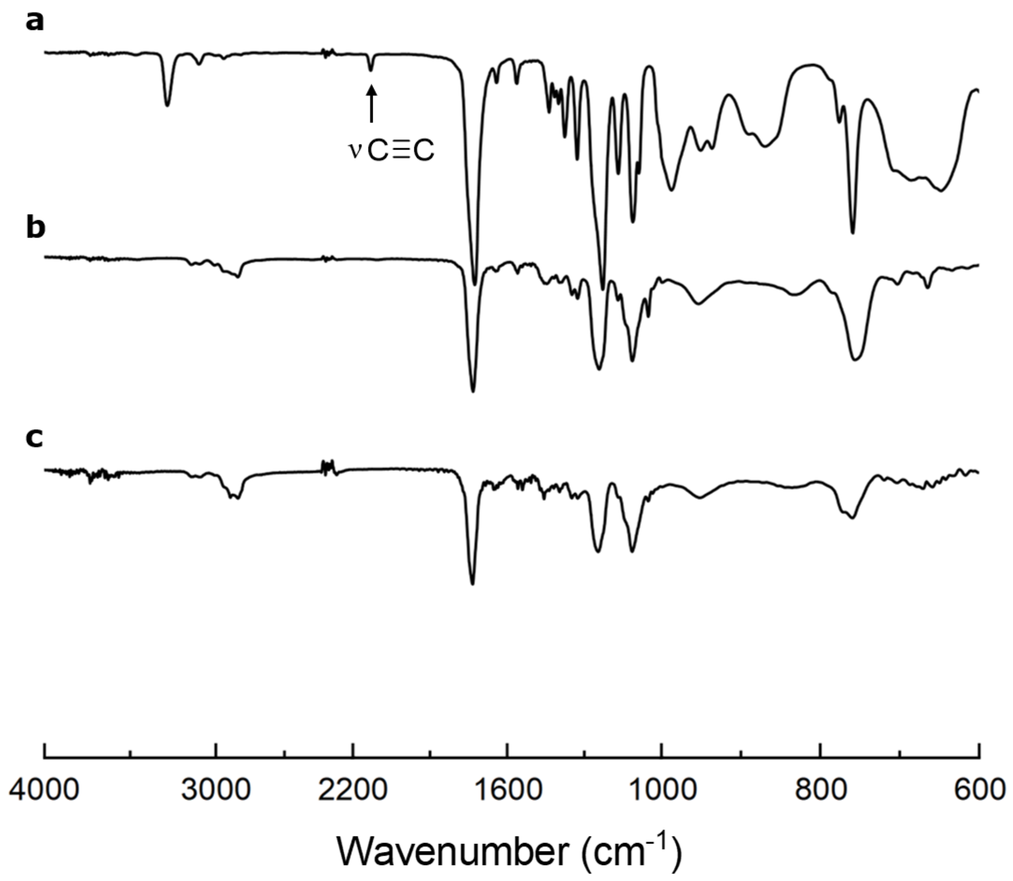

Figure S4. Infrared spectra of (a) **1**, (b) **P1**, and (c) **P2**.

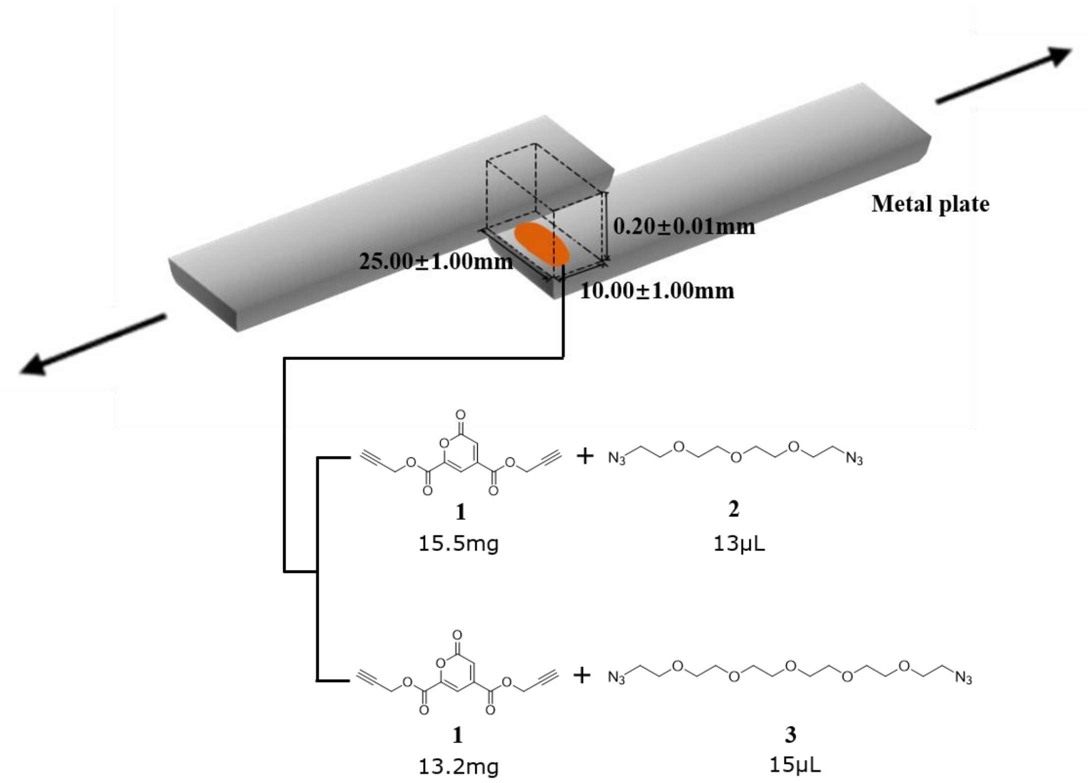

Figure S5. The tensile lap-shear strengths measurements of the in-situ polymerized **P1** (**1**+**2**) and **P2** (**1**+**3**).

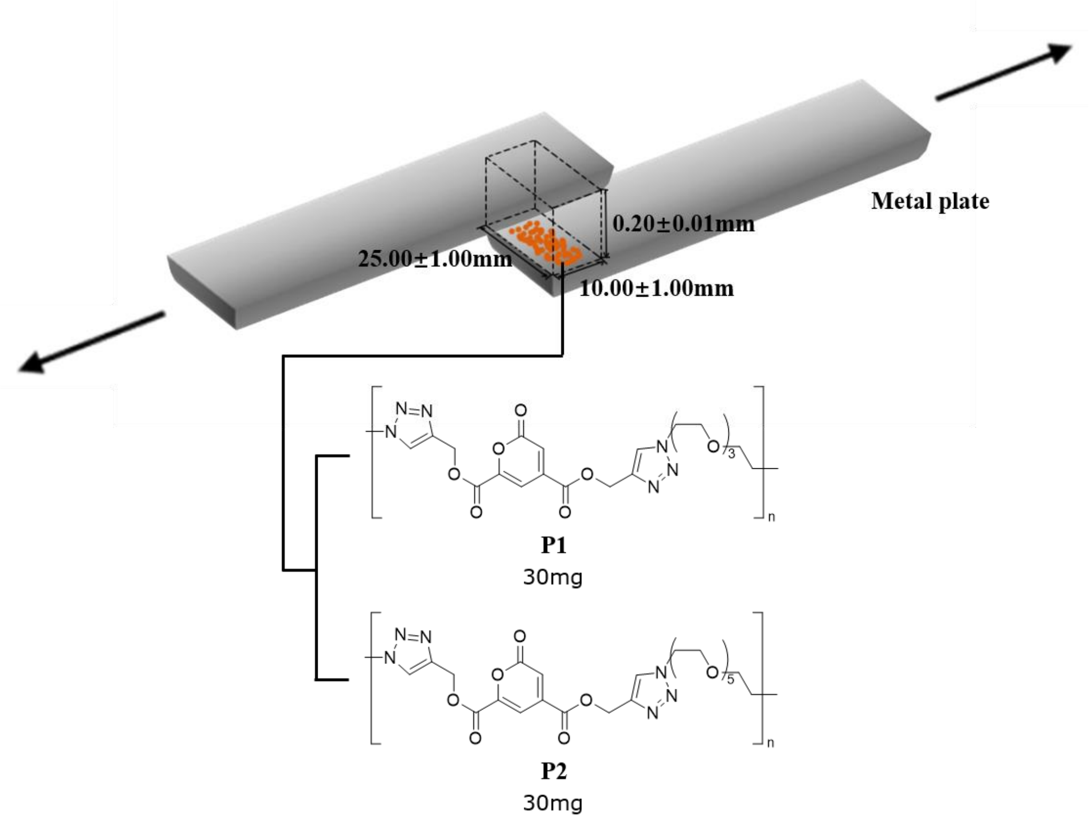

Figure S6. The tensile lap-shear strengths measurements of **P1** and **P2** prepared by CuAAC polymerization.
